# Bilateral congenital inguinal hernia with right-sided Amyand's hernia in a premature twin: Case report and a summary of clinical presentations, management and outcomes in neonates and infants with Amyand's hernia

**DOI:** 10.1016/j.ijscr.2021.106505

**Published:** 2021-10-14

**Authors:** Urías De Jesús Hernández-López, Audrey Vargas-Buelvas, Willfrant Jhonnathan Muñoz-Murillo, Katherine Lizeth Muñoz-Murillo, Gian Nuñez-Rojas, Sabrina Rahman

**Affiliations:** a Department of Medicine, Universidad de Cartagena, Cra. 50 #24-120, Cartagena, Colombia; b Department of Surgery, Universidad de Cartagena, Cra. 50 #24-120, Cartagena, Colombia; c School of Medicine, Universidad del Quindío, Carrera 15 #12N, Armenia, Colombia; d Medical and Surgical Research Center, School of Medicine, Universidad de Cartagena, Cra. 50 #24-120, Cartagena, Colombia; e Department of Public Health, Independent University-Bangladesh, Dhaka, Bangladesh

**Keywords:** Amyand's hernia, Inguinal hernia, Premature birth, Congenital hernia, Twins

## Abstract

**Introduction and importance:**

Congenital hernias occur 70% on the right side, 25% on the left side, and approximately 5% bilaterally. The finding of a congenital Amyand's hernia is of interest, especially in patients who do not present risk factors associated with connective tissue disorders, ascitic conditions, fetal developmental disorders or any condition that increases abdominal pressure.

**Case presentation:**

Male patient, 6 months old, was brought to the pediatric surgery department due to a visible mass in the bilateral inguinal region, which protruded with crying. The parents report that he was a 36-week preterm, low birth weight, monochorionic monoamniotic twin with bilateral congenital inguinal hernia. An open herniorrhaphy was performed, showing a left communicating hydrocele with an indirect left inguinal hernia and right communicating hydrocele with indirect inguinal hernia containing cecal appendix with no signs of inflammation.

**Clinical discussion:**

The most common clinical presentation is the presence of a reducible or irreducible mass, erythema and/or inguino-scrotal edema, irritability manifested by crying and recurrent pain in older infants. This condition may be associated with cryptorchidism, intrauterine structural developmental disorder, and the presence of fistulas. Appendectomy and traditional hernia reduction are the most common surgical approach. The evolution of this condition is favorable with extremely low complication rates.

**Conclusion:**

Amyand's hernia in the neonate is a rare presenting condition, which frequently involves nearby structures with risk of inflammation, incarceration and perforation, so repair should be performed early.

## Highlights

• Currently there are controversies in the approach of congenital inguinal hernias.
• The overall outcome in the management of congenital hernia is favorable.
• Traditional hernia management for congenital Amyand's hernia has good results.

## 1 Introduction

Inguinal hernia is one of the most common surgical conditions evaluated by surgeons around the world in all age groups, estimating more than 800.000 inguinal repairs annually in the United States [1]. This type of hernia accounts for approximately 75% of abdominal wall hernias. Its etiology can be congenital or acquired, the acquired hernia being the most frequent in adults. Ninety percent of hernias occur in men, with a bimodal peak at 5 and 70 years of age [1]. Unlike in adults, the most frequent etiology in neonates is congenital, with an incidence of 3% in newborns born at term and 13% in preterm infants less than 33 weeks [2]. It has been described that congenital inguinal hernias occur due to a failure in the closure of the patent processus vaginalis at the level of the deep inguinal ring, precipitating the passage of intra-abdominal contents through the inguinal canal and ring, or scrotum in men and via the canal of Nuck to the labium in women [3].

Congenital hernias occur 70% on the right side, 25% on the left side, and approximately 5% bilaterally [3]. On the other hand, the finding of a congenital Amyand's hernia is of interest, especially in patients who do not present risk factors associated with connective tissue disorders, ascitic conditions, fetal developmental disorders or any condition that increases abdominal pressure [3], [4]. More exceptionally, a bilateral congenital inguinal hernia with associated Amyand's hernia. This situation represents a challenge due to the lack of evidence regarding the preoperative considerations, the time from diagnosis to intervention and the surgical technique to be used [5]. Some of the recommendations in the management of inguinal hernia in the neonate is that it should be resolved surgically immediately after diagnosis to avoid incarceration and testicular atrophy [2]. However, there is no evidence of the highest quality on the time-outcome relationship in congenital inguinal hernia repair. Therefore, some of the current controversies regarding congenital inguinal hernia repair are the role of laparoscopy, overnight admission for apnea monitoring, contralateral exploration in those with unilateral hernia and the optimal timing of surgical repair [5].

Taking into account this evidence gap, the objective of this case is to report an interesting case of bilateral congenital inguinal hernia associated with right-sided Amyand's hernia in a premature twin, and to summarize the clinical presentation, management and outcomes of the few cases reported on neonates and infants with congenital Amyand's hernia. This case report followed the SCARE guidelines for its realization [6].

For the summary of clinical presentations, management and outcomes in neonates and infants with Amyand's hernia, a non-systematic search of the literature was performed in the PubMed database over a period of 16 years (2005–2021), with the key terms “Amyand's hernia” and “Neonate” and synonyms, together with the Boolean operator AND/OR, with the aim of gathering the largest number of related articles; finally obtaining 29 articles. Articles consisting of original studies, case reports, and case series were included. We excluded those articles that did not present data on clinical presentations, management and outcomes of Amyand's hernia, as well as those that did not have full text available. After the application of these criteria, 15 studies were finally included **[**Table 1**]** [4], [7], [8], [9], [10], [11], [12], [13], [14], [15], [16], [17], [18], [19], [20].

**Table 1:** Summary of selected studies reporting clinical presentations, management and outcomes in neonates and infants with Amyand's hernia.

Authors	Number and age of patients evaluated	Clinical presentation	Management	Outcomes
Mohamed et al. [4]	1 patient / 19 days of birth	The neonate was taken to the emergency room with a clinical history of inguino-scrotal erythema and persistent irritability; physical examination revealed a distended, soft and painless abdomen and bilateral scrotal edema.	Exploratory laparotomy was performed to exclude any source of intra-abdominal sepsis, and to allow adequate surgical access to treat the possibility of a hernia. The appendix was found to be incarcerated and inflamed, and an appendectomy was performed	The inguinoscrotal erythema resolved and the patient was discharged home
Omran et al. [7]	1 patient / 28 days of birth	History of right-sided infection and edema of the spermatic cord after 14 days, admitted to the emergency room for increased swelling in the right scrotum, physical examination revealed large inguinal scrotal swelling	Ultrasonography reported right testicular torsion. Exploration was performed through an incision in the lateral fold to the pubic tubercle, observing the fascia of the spermatic cord, the right testicle was removed and the pus of the spermatic cord was drained.	The postoperative course was uneventful and the patient was discharged home after 5 days
Yodoshi et al. [8]	1 patient / 10 days of birth	The neonate was admitted to the emergency room with irritability, temperature of 37.9° C and erythema in the right inguinal region. Imaging studies were performed, but there were no findings, so he was discharged with a diagnosis of acute epididymitis. The patient returned 6 h later to the emergency room with fever and extreme erythema of the scrotum	Abdominal radiography showed gas in the right hemiscrotum. The indication for surgery was for incarcerated hernia. Intraoperative findings revealed inguinal hernia with attached appendix, and appendectomy was performed	His hospital course was excellent, with no complications, and he was discharged home 3 days after surgery
Fascetti-León et al. [9]	1 patient / 29 days of birth	The neonate was admitted to the emergency department with a 3-h history of right-sided groin swelling. Physical examination revealed swelling of the right groin and the testicle was palpated separately. The diagnosis of the undescended right testicle had been made 4 days earlier	A diagnosis of irreducible inguinal hernia was made days later. The patient underwent laparoscopy, which revealed dilated bowel loops and an inflammatory mass in the right iliac fossa, inflamed and necrotic appendix. Conversion to laparotomy was made and the appendectomy was performed	The postoperative course was normal without complications and the patient was discharged on the third day
Erginel et al. [10]	1 patient/ 24 days of birth	The neonate developed scrotal swelling at 24 days of life, two days later he was brought to the clinic, physical examination revealed hard right testicle, local heat and erythema	Inguino-scrotal ultrasound suggested testicular ischemia and an edematous right testicle, so incarcerated inguinal hernia was suspected. Emergency surgery was performed and a right transverse incision was made, finding thickened spermatic cord and incarcerated perforated appendix, in addition to purulent fluid	The patient was discharged home on the fifth day without complications
Cigsar et al. [11]	4498 patients. 46 patients had amyand's hernia, being all males with a mean age of 16.7 months	On physical examination, groin swelling was the most common finding, followed by tenderness, pain, fever and vomiting	Thirty-seven (80.4%) right, two (4.3%) left and seven (15.2%) bilateral hernioplasties were performed. Nine patients underwent emergency surgery with an initial diagnosis of incarcerated hernia, with amyand's hernia being an incidental finding in the remaining 37 patients	Surgical findings included 33 normal appendices, 9 inflamed appendices, one perforated appendix and three appendices attached to the hernial sac; no patient developed recurrent hernia or appendicitis during the follow-up period.
Mandhan et al. [12]	1 patient / 5 days of birth	The neonate was referred with right inguino-scrotal swelling, physical examination revealed right inguino-scrotal swelling with mild erythema, in addition to dysmorphic features along with bilateral central cleft lip and cleft palate, bilateral postaxial polydactyly and clenched fists	Initial ultrasound findings suggested the diagnosis of right epididymo-orchitis and antibiotic management was initiated. 34–48 h later, the right inguino-scrotal swelling worsened, so he underwent a surgical exploration with an inguinal incision, finding firm swelling in the right scrotum and a twisted and gangrenous appendix	The patient was discharged without complications
Sandhu et al. [13]	1 patient / 5 days of birth	The neonate presented right inguino-scrotal swelling for 4 days with a history of bilious vomiting and progressive abdominal distension, on physical examination he was febrile, with signs of dehydration and irreducible painful swelling	Abdominal X-ray showed multiple hydro-aerial levels, making a diagnosis of obstructed inguinal hernia. An incision was made in the inguinal skin fold, finding purulent material on opening the sac and perforated appendix, appendectomy and high ligation herniotomy were performed.	The patient's postoperative recovery remained uneventful
Sun et al. [14]	1 patient / 24 days of birth	The neonate was taken to the pediatric surgery department with swelling of the scrotum for 4 days, abdominal distension, physical examination revealed a firm and tender swelling in the right inguinal region	The ultrasound detected a colon echo in the right inguinal canal, showing hyperechoes in movement in the lumen, for which a diagnosis of right inguinal hernia and right encapsulated hydrocele was made. Surgical exploration was performed revealing swollen tunica vaginalis of the right testicle and purulent discharge. The colon-like structure was identified as appendix	The postoperative period was normal without complications. Ultrasound was performed one week later and revealed normal scrotum and testicles
Panagidis et al. [15]	1 patient / 25 days of birth	The neonate was referred to the institution for entero-cutaneous fistula with fecal discharge from the right hemiscrotal. On physical examination he was septic, febrile, with a heart rate of 170 beats/min, mottled skin on the trunk and extremities, edematous penis and scrotum.	An abdominal X-ray was performed, showing distended enteric loops and presence of gas in the scrotum. A strangulated inguinal hernia was diagnosed, complicated by perforation of the intestine and formation of an entero-cutaneous fistula. An inguinal incision was made and a perforated vermiform appendix was found in the scrotum	The postoperative hospital stay was normal, scrotal Doppler ultrasound was performed 3 months and 6 months after surgery, the right testicle was viable and the hernia did not reappear
Park et al. [16]	1 patient / preterm of 30 weeks and 33 days of birth	The neonate was taken to the emergency room with swelling and erythema of the right hemiscrotum. Physical examination revealed a reducible left inguinal hernia and a non-reducible right scrotal mass. The erythema extended to the inner thighs, perineum and anterior abdominal wall	The initial scrotal Doppler ultrasound showed increased flow in the right testicle, epididymis and inguinal canal. Fluid accumulation was observed in the right testicle, and the CT scan showed gas and fluid collection in the testicle, the patient was scheduled for diagnostic laparoscopy and proceeded to perform appendectomy and bilateral inguinal hernia repair	The patient tolerated the procedure well, evolved satisfactorily and was discharged from the neonatal ICU at 49 days of life
Ngom et al. [17]	1 patient / 14 days of birth	He was referred to the service for right inguino-scrotal swelling associated with excessive crying without vomiting or alterations in bowel habits. On physical examination the patient was found in good general condition, fever and painful and irreducible right inguino-scrotal mass.	The preoperative diagnosis was strangulated inguinal hernia. Surgery was performed with an inguinal approach discovering a perforated appendix inside the hernia sac, and appendectomy was performed	The postoperative period passed without complications, the patient was followed up for 1 year, observing the absence of symptoms
Upadhyaya et al. [18]	1 patient / 7 months of age	History of recurrent pain in the right inguinal region intermittently	He underwent elective surgery. On examination, the appendix was observed adhered to the hernial sac, so appendectomy was performed and the sac was ligated	The patient was discharged without complications on postoperative day 4 and remained in optimal condition during follow-up
Esposito et al. [19]	1 patient / 1 month of birth	The neonate was brought to the emergency room with an incarcerated left inguinal hernia, physical examination revealed a small non-communicating hydrocele on the right side	Manual reduction was unsuccessful, so it was decided to perform a laparoscopic exploration showing evidence of left hernia with intestinal loops, and on the right side appendix, completely incarcerated in the hernial sac. The appendix was dissected from the sac and then reduced to the abdomen	The patient was reoperated two days later because he presented fever and abdominal distension. The surgery was performed by pfannenstiel incision and the ischemic appendix was removed. The postoperative course was uneventful.
Ergaz et al. [20]	1 patient / preterm neonate 32 weeks, 30 days of birth	At 30 days of age, on routine physical examination, the right scrotum was hard and swollen, afebrile	Abdominal X-ray showed dilated bowel loops, and abdominal ultrasound showed a looped and hyperemic testicle in a hyperemic thickened scrotum, with a presumptive diagnosis of incarcerated inguinal hernia. An incision was made in the right region, where an inflamed appendix was evidenced. Appendectomy and herniotomy were performed	The postoperative course was normal, the patient was discharged after 36 days and was followed up at 2 months with a normal physical examination

## 2 Presentation of case

Male patient, 6 months old, was brought to the pediatric surgery department due to a visible mass in the bilateral inguinal region, which protruded with crying. Parents report no changes in skin coloration or size changes over time in a resting condition. The parents also report that he was a 36-week preterm, low birth weight (2400 g), monochorionic monoamniotic twin with bilateral congenital inguinal hernia. During the first days of his birth, he developed bronchiolitis which was treated and completely resolved. During the mother's pregnancy there were no complications, it was a controlled pregnancy, the delivery was vaginal and the other twin did not present any morbidity or perinatal complication. Considering he was a patient at risk, the neonatology department decided to wait a few days to evaluate the management of the bilateral inguinal hernia. However, due to family issues, the parents did not attend until the sixth month after birth.

**Fig. 1:**
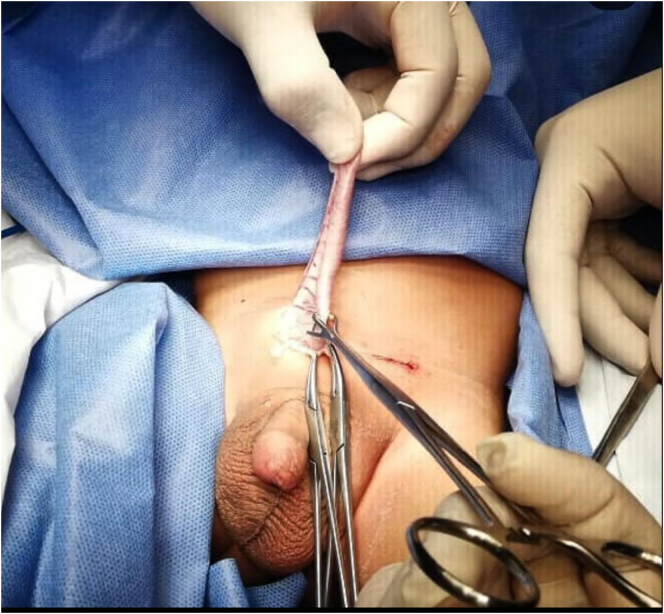
Intraoperative photograph showing the cecal appendix exteriorized by the external inguinal ring.

Physical examination confirmed the presence of bilateral movable inguinal masses of semi-soft consistency, which protrudes with Valsalva manipses. Considering the history of the diagnosis of bilateral congenital inguinal hernia, it was decided to perform elective open herniorrhaphy, according to hospital protocol. This procedure is performed with high sac ligation, distal hydrocelectomy and appendectomy with invagination of the stump. During the operation, left communicating hydrocele with indirect left inguinal hernia, and right communicating hydrocele with indirect inguinal hernia containing cecal appendix with no signs of inflammation were evidenced **(**Fig. 1**)**.

The patient underwent the postoperative period without complications and was discharged after 5 days. The patient returned to the outpatient clinic 15 days later, where the surgical wound with scar tissue was observed, with no signs of acute inflammation or secretions. The parents were satisfied with the approach and the results of the patient.

## 3 Discussion

As reported in the literature, the average age at which surgical repair of Amyand's hernias is performed during the neonatal and infancy period ranges from 5 days after birth to 16 months [7], [8], [9], [10], [11], [12], [13], [14], [15], [16], [17], [18], [19], [20]. The most common clinical presentation is the presence of a reducible or irreducible mass, erythema and/or inguino-scrotal edema, irritability manifested by crying and recurrent pain in older infants [7], [8], [9], [10], [11], [12], [13], [14], [15], [16], [17], [18], [19], [20]. This condition may be associated with cryptorchidism [9], intrauterine structural developmental disorder [12], and the presence of fistulas [15]. In case of a complicated hernia, it may be accompanied by progressive abdominal distension, vomiting and fever [13]. One of the most common differential diagnoses is acute epididymitis [8].

The most commonly used imaging techniques for the evaluation of hernia are ultrasonography (which can demonstrate the presence of torsion or testicular ischemia) [7], [10], [12], [14], [16], and abdominal radiography (which demonstrates the presence of gas in the case of an incarcerated hernia) [8], [13], [15], [20]. Although many cases are initially operated on laparoscopically, the finding of a necrotic appendix with inflamed loops is one of the main conversion factors for laparotomy, as well as the compromise of abdominal structures such as the intestinal loops [9]. Appendectomy and traditional hernia reduction are the most common surgical approach [7], [8], [9], [10], [11], [12], [13], [14], [15], [16], [17], [18], [19], [20]. Most of the cases analyzed were associated with inflammation, incarceration or appendiceal perforation with the presence of purulent discharge [4], [7], [9], [10], [11], [12], [13], [15], [16], [17], [19], [20] and, interestingly, age is not a significant associated factor, since these findings are found in patients who are from a few days old to several weeks old.

Of the cases evaluated, absolutely all had a satisfactory postoperative course without complications or recurrence [4], [7], [9], [10], [11], [12], [13], [14], except for the case of Esposito et al. [19], who dissected the hernia sac and returned the appendix to the abdominal cavity, but had to reoperate two days later due to the development of appendicitis. Therefore, it can be presumed that in all cases the cecal appendix should be resected to avoid future complications. Particularly, it is observed that most of the cases presented incarceration of the hernia with inflammation of nearby structures [4], [7], [9], [10], [11], [12], [13], [15], [16], [17], [19], [20]. In other words, the ideal would be to perform the hernia repair soon after diagnosis, in order to reduce the risk of appendiceal perforation and progression to sepsis, as in the case of Panagidis et al. [15].

Although in this case the approach was made at 6 months of age, in spite of having been diagnosed a few days after birth, there was no evidence of a perforated or inflamed cecal appendix, only the development of hydrocele. In contrast to the manifestations commonly reported in the literature, such as skin erythema, in the present case only the protrusion of the mass was evidenced by crying. The approach was exploratory laparotomy due to the lack of training in the use of laparoscopy in pediatric surgery in low- and middle-income countries, such Colombia. However, the postoperative course was satisfactory. More studies of the highest quality are needed to determine the factors associated with the development of complications and to establish a cut-off time for surgical repair in cases of Amyand's hernia in neonates and infants.

As a limitation, due to organizational difficulties it was not possible to obtain more intraoperative photographs, nor was there access to the initial diagnostic images during the perinatal period, due to loss of this data by the parents. Finally, the parents understood her baby's condition and was satisfied with the approach and effort made by the medical team. In contrast to what is currently published in the literature, this manuscript summarizes clinical presentations, management and outcomes in neonates and infants with Amyand's hernia and describes the presentation of a rare case of bilateral congenital inguinal hernia associated with uncomplicated Amyand's hernia.

## 4 Conclusion

Amyand's hernia in the neonate is a rare presenting condition, which frequently involves nearby structures with risk of inflammation, incarceration and perforation, so repair should be performed early. Ultrasonography and abdominal radiography are the imaging techniques of choice to evaluate the inguinal and abdominal region, and to rule out any other differential diagnosis or associated complications such as bowel loop perforation, testicular torsion or hydrocele. Appendectomy and traditional reduction are approaches with a favorable yield, and the appendix should always be resected to avoid the development of appendicitis later on. The evolution of this condition presents extremely low complication rates.

## Sources of funding

Non declared.

## Ethical approval

Hospital exempts ethics approval for reported cases.

## Consent written

Written informed consent was obtained from the patient for publication of this case report and accompanying images. A copy of the written consent is available for review by the Editor-in-Chief of this journal on request.

## CRediT authorship contribution statement

All authors equally contributed to the analysis and writing of the manuscript.

## Research registration

Not applicable.

## Guarantor

Sabrina Rahman. Department of Public Health, Independent University-Bangladesh, Dhaka, Bangladesh. sabrinaemz25@gmail.com

## Provenance and peer review

Not commissioned, externally peer-reviewed.

## Declaration of competing interest

The authors declare that they have no known competing financial interests or personal relationships that could have appeared to influence the work reported in this paper.
